# To Keep Apples

**Published:** 1878-08

**Authors:** 


To Keep Apples.—" The most effectual method of preserving both apples and pears with which I am familiar—and which of course I recommend in preference to all others, is the following: Having selected the best fruit, wipe it perfectly clean and dry with a fine cloth, then take a jar of suitable size, the inside of which is thoroughly coated with cement, and having placed a layer of fine sand perfectly dry at the bottom, place thereon a layer of the fruit—apples or pears as the case may be—but not so close as to touch each other, and then a layer of sand; and in this way proceed till the vessel is full. Over the upper layer of fruit a thick stratum of sand may be spread and lightly pressed down with the hands. In this manner choice fruit perfectly ripe may be kept for almost any length of time, if the jar be placed in a situation free from moisture."

